# Molecular level computational studies of polyethylene and polyacrylonitrile composites containing single walled carbon nanotubes: effect of carboxylic acid functionalization on nanotube-polymer interfacial properties

**DOI:** 10.3389/fchem.2014.00074

**Published:** 2014-09-02

**Authors:** Shayesteh Haghighatpanah, Martin Bohlén, Kim Bolton

**Affiliations:** School of Engineering, University of BoråsBorås, Sweden

**Keywords:** polyethylene, polyacrylonitrile, carbon nanotubes, interface, functionalization, pull-out, molecular mechanics, molecular dynamics

## Abstract

Molecular dynamics (MD) and molecular mechanics (MM) methods have been used to investigate additive-polymer interfacial properties in single walled carbon nanotube (SWNT)—polyethylene and SWNT—polyacrylonitrile composites. Properties such as the interfacial shear stress and bonding energy are similar for the two composites. In contrast, functionalizing the SWNT with carboxylic acid groups leads to an increase in these properties, with a larger increase for the polar polyacrylonitrile composite. Increasing the percentage of carbon atoms that were functionalized from 1 to 5% also leads to an increase in the interfacial properties. In addition, the interfacial properties depend on the location of the functional groups on the SWNT wall.

## Introduction

There has been great interest in carbon nanotubes (CNTs) since their discovery in 1991 (Iijima, 1991), primarily due to their unique mechanical, electrical and thermal properties. For example, the superior strength of CNTs combined with their light weight makes them suitable as reinforcement additives in polymeric nanocomposites (PNCs) (Lourie and Wagner, 1998; Schadler et al., 1998; Wagner et al., 1998; Cooper et al., 2002; Barber et al., 2003; Gou et al., 2004). The properties of the CNTs, the polymer and the interface between these materials determine the properties of the PNCs. In this regard, one of the important aspects is the interfacial bonding between the CNTs and the polymer matrix, since a stronger interfacial bonding results in an improved load transfer from the polymer matrix to the nanotubes (Gou et al., 2004).

Proper dispersion of CNTs in the polymer matrix, which is hindered by the strong van der Waals (vdW) interactions between the CNTs, is also an important challenge when optimizing PNC properties (Jin et al., 1998; Qian et al., 2000). The dispersion is expected to improve when the CNT-polymer interactions are sufficiently strong to compete with the CNT-CNT bonding. Good dispersion of CNTs has been reported in polar polymers such as polyacrylonitrile (Chae et al., 2005). However, CNT dispersion in nonpolar polymers, such as polypropylene (PP), during melt processing remains a challenge (Girei et al., 2012). Two approaches have been proposed to achieve improved dispersion of CNTs and a strong interfacial bonding at the interface between CNTs and the polymer matrix. The first approach is non-covalent (physical) interactions and/or wrapping of polymers on the surface of the CNTs, and the second is covalent functionalization of the CNTs (Ge et al., 2004; Spitalsky et al., 2010).

Polyethylene (PE) is a polymer that is used to make many products, such as plastic films and sheets, a wide variety of containers, kitchenware and tubing (Cheremisinoff, 1996). It is the most widely studied and commercially used polymer. The types of PE vary depending on the method of manufacture and the amount and type of co-monomer, which can contain a hydrocarbon or a polar group (Tewarson, 2007). Polyacrylonitrile (PAN) is another polymer that is of great interest due to its commercial and technological uses (Masson, 1995), good stability and mechanical properties. PAN is also used as carbon fiber precursors (Nataraj et al., 2012). It has applications in areas such as electronics, tissue engineering, membrane filtration, and high performance composites (Nataraj et al., 2012).

Several experimental studies have evaluated the mechanical properties of PNCs (Lourie and Wagner, 1998; Schadler et al., 1998; Wagner et al., 1998; Cooper et al., 2002; Barber et al., 2003). For example, Wagner et al. (1998) showed that the interfacial shear stress, τ, between multi-walled carbon nanotubes (MWNTs) and polymer matrices (urethane/diacrylate oligomer EBECRYL 4858) under compression and strain can be as high as 500 MPa. Qian et al. (2000) reported that 1 wt.% of MWNTs in polystyrene (PS) composites increases the tensile modulus and tensile strength by 36–42% and 25%, respectively. This indicates a high load transfer in the composites. Raman spectroscopy experiments by Ajayan et al. (2000) showed that there is poor interfacial load transfer in single walled carbon nanotube (SWNT)—epoxy composites. Cooper et al. (2002) attempted to measure the interfacial strength by pulling out individual SWNT ropes and MWNTs from an epoxy matrix using a scanning probe microscope tip. Although they found that the shear stress at the MWNT-epoxy interface is in the range of 35–376 MPa, most of SWNT ropes were fractured instead of being pulled-out from the epoxy matrix. In an atomic force microscopy study, Barber et al. (2003) reported that 47 MPa is required to pull out MWNTs from a polyethylene-butene matrix.

Chae et al. (2005) studied CNT-PAN composites and found a maximum increase in modulus of 75% in SWNT-containing composites, and a maximum improvement in tensile strength of about 70% in composites containing MWNTs. Ge et al. (2004) electrospun highly oriented, large area continuous composite nanofiber sheets made of surface-oxidized MWNTs and PAN. Tunneling electron microscopy and electron diffraction measurements showed that the preferred orientation of the MWNTs was along the fiber axis. They showed that charge transfer complexes formed between surface-oxidized nanotubes and the negatively charged functional groups in PAN during electrospinning led to strong interfacial bonding between the CNTs and the surrounding polymer chains. They also found that the tensile modulus of the compressed composite nanofiber sheets was improved significantly to 10.9 and 14.5 GPa along the fiber winding direction at MWNT loadings of 10 and 20 wt.%, respectively. Weisenberger et al. (2003) studied the suitability of MWNTs as a reinforcing filler in a PAN matrix with the goal of producing composite fibers containing MWNTs. The MWNTs were dispersed in a PAN—dimethylacetamide (DMAc) solution, and composite fibers were spun using a dry-jet wet spinline, which resulted in axial alignment of the MWNTs. Tensile measurements on individual fibers showed significant enhancement in mechanical properties compared to the raw PAN fibers, including increases of 31% in break strength, 36% in modulus and 46% in yield strength at a loading of 1.8 vol.% MWNT.

Computational studies complement experiment by providing easy manipulation and analysis at the molecular level. For example, due to the difficulties of experimental manipulation at the nano-scale, it has been difficult to develop a method to measure the interfacial strength between CNTs and the polymer matrix. There are several computational techniques that can provide this data. First principles methods are able to generate reasonably accurate data of structures and energies relevant to PNC systems, but they can only be used to study small systems over short times due to their computational expense. In contrast, molecular simulation methods such as molecular mechanics (MM) and molecular dynamics (MD) that are based on analytic force fields are computationally cheaper. They can therefore be used to study larger molecular systems for longer times. As described below, MD can also be used to obtain macroscopic properties such as the interfacial shear stress. The validity of the results obtained from force field based methods is dependent on the analytic function used for the force field as well as its parameterization. Therefore, it is necessary to use a force field which correctly describes the dynamics and trends of the system and properties under investigation. The validity of the force field is often ensured by comparison with first principles and/or experimental data (van Gunsteren and Berendsen, 1990).

Force field based methods have been widely used to study PNCs. For example, geometry optimization (GO) and MD methods have been used to understand the additive-polymer bonding characteristics and to predict the interfacial shear stress and mechanical properties at the additive-polymer interface (Lordi and Yao, 2000; Liao and Li, 2001; Frankland et al., 2002, 2003; Wong et al., 2003; Yang et al., 2005; Mokashi et al., 2007; Natsuki et al., 2007). MD simulations by Li et al. (2011) showed that, for SWNT-PE composites, the pull-out force is independent of nanotube length and is proportional to the nanotube diameter. They found that the interfacial shear stress initially decreases with increasing nanotube diameter and finally saturates at a value of 107 MPa when the diameter is ~10 nm. They also found that the surface energy density has the same trend as the interfacial shear stress and converges to 0.11 N/m. In agreement with this, Haghighatpanah and Bolton (2013) found an interfacial shear stress and surface energy density of SWNT-PE structures of 141 MPa and 0.14 N/m. Zheng et al. (2007) also used MD to study the interactions between SWNTs and several polymers (i.e., PE, PS, polypropylene and polyaniline), when the polymer was either wrapped around the SWNT or inside the SWNT. They found that the interaction strength depends on the monomer structure (e.g., aromatic rings) and the nanotube chirality and diameter. MD simulations by Frankland et al. (2002) showed that the load transfer and modulus of SWNT-polymer composites could be increased by addition of chemical cross-linking between the nanotubes and polymer matrix. Hence, inadvertent creation of SWNT-polymer covalent bonds during processing may be partially responsible for enhanced stress transfer observed in some systems. Zheng et al. (2008) studied the influence of chemical functionalization on the interfacial bonding characteristics of SWNTs reinforced polymer composites using MM and MD simulation. They showed that functionalization of nanotubes at low densities of functionalized carbon atoms drastically increases their interfacial bonding strength and the shear stress between the nanotubes and the polymer matrix. Functionalization of as little as 5.0% of the nanotube carbon atoms increases the shear stress by about three orders of magnitude.

This contribution reports additive-polymer interfacial properties obtained from pull-out simulations using MM and MD. SWNTs were used as the additive and PE and PAN were used for the polymer matrices. In addition to the importance of these polymers described above, they also represent non-polar and polar polymers, respectively. The effect of SWNT functionalization was studied by adding carboxylic acid functional groups, –COOH, to the SWNT. These groups were added to 1 and 5% of the SWNT atoms, which is typical for experimental investigations (Girei et al., 2012; Wang et al., 2012). In addition, carboxylic acid functionalization is very attractive since it can be readily used for further covalent and non-covalent functionalization of SWNTs (Niyogi et al., 2002). This is the first molecular-level study of functionalized SWNT-PE and SWNT-PAN composites, and focuses on the effect that functionalization and polymer type (non-polar or polar) have on the trends of additive-polymer interfacial properties.

## Methods

### Force field

The Condensed-phase Optimized Molecular Potentials for Atomistic Simulation Studies (COMPASS) force field (Sun, 1998) was used for this study. This is an all-atom force field that has been developed for common organic molecules, inorganic small molecules and polymers. The parameters are fit to both first principles and experimental data. The valence parameters (including diagonal and off-diagonal cross-coupling terms) and atomic partial charges are fit to ab initio data. The vdW parameters are derived from MD simulations of molecular liquids and by fitting the simulated cohesive energies and equilibrium densities to experimental data (Sun, 1998). Our previous study (Haghighatpanah and Bolton, 2013) showed that any of the Dreiding, Universal or COMPASS force fields provide a valid description of SWNT-PE composites. However, the COMPASS force field is preferred over the Dreiding (Mayo et al., 1990) and Universal (Rappe et al., 1992) force fields for systems containing oxygen atoms (cellobiose was studied in the previous work) (Bazooyar et al., 2012), and is therefore used in the present study of the effect of carboxylic acid functionalization of SWNTs.

### SWNT pull-out

#### Non-functionalized SWNTs

**Figure 1:**
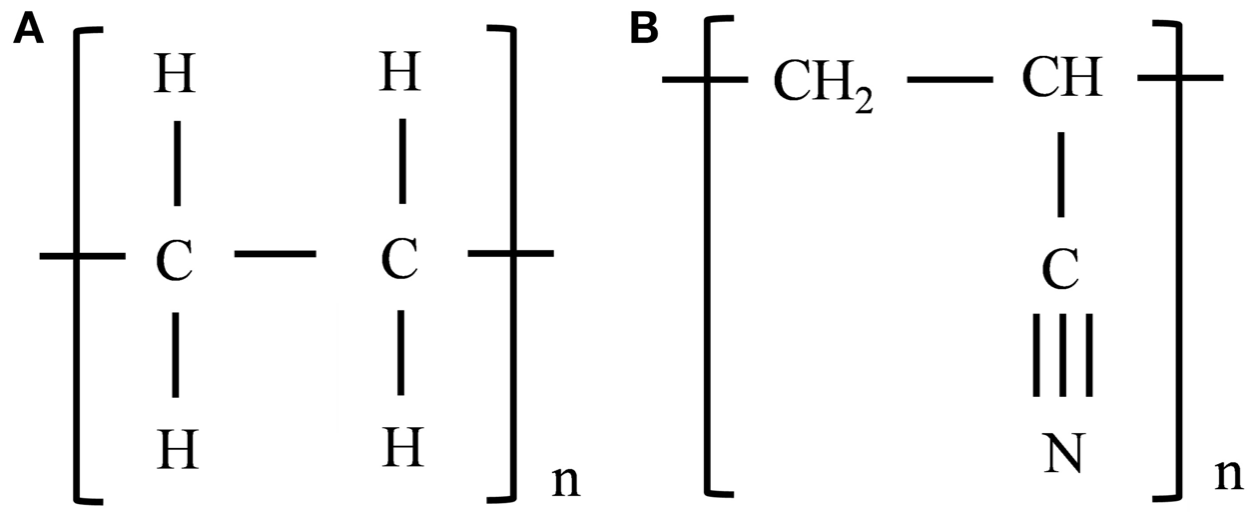
**Illustration of repeat units of PE (A) and PAN (B) polymer chains**.

Three systems were constructed for each of the SWNT-polymer nanocomposites studied here. The similarity of the results obtained from each of the three systems, and the consistent trends obtained when increasing the extent of SWNT functionalization, shows that this is a sufficient number to obtain statistically converged results. Each system was constructed by placing the SWNT at an edge of a periodic cell (to allow for their extraction once the periodic boundary conditions are removed) and then randomly placing the polymer chains around the SWNT (where each chain had a randomly chosen amorphous structure). The SWNT, which was an armchair (5,5) nanotube terminated with hydrogen atoms at both ends, was 44 Å long. This is sufficiently long to obtain converged results (Li et al., 2011). It may be noted that the SWNT chirality may affect the SWNT-polymer interaction strength, but these effects are expected to be minor compared to the effect of carboxylic acid functionalization discussed below (and the trends obtained with carboxylic acid functionalization of the (5,5) SWNT are expected to be valid for all chiralities). The SWNT was surrounded by 73 PE chains composed of 15 –CH_2_–CH_2_– repeat units in the SWNT-PE composite and 50 PAN chains composed of 15 –CH_2_–CH–CN– repeat units in the SWNT-PAN composite. Previous studies have shown that these chains are sufficiently long to obtain converged results (Zheng et al., 2009; Li et al., 2011; Haghighatpanah and Bolton, 2013), and the number of chains was selected to obtain the correct densities of the amorphous systems (Hurley and Tzentis, 1963; Vasile and Pascu, 2005). The repeat units of PE and PAN chains are shown in Figures 1A,B, respectively.

The periodic SWNT-PE and SWNT-PAN amorphous cells were constructed with volumes of 33.17 Å × 33.17 Å × 71.54 Å and 33.42 Å × 33.42 Å × 71.54 Å, respectively. This yields a SWNT volume fraction of ~5%, which is in the range of volume fractions used in experiments (Cebeci et al., 2009; Girei et al., 2012). The cells are also sufficiently large to prevent interaction between atoms and their periodic images. In addition, as discussed previously (Haghighatpanah and Bolton, 2013), similar changes in interaction energy during pull-out have been calculated by other authors (Haghighatpanah and Bolton, 2013), which shows that the results are not sensitive to the different box sizes used in the calculations (although they depend on the length and diameter of the SWCNT). The density of each SWNT-PE and SWNT-PAN system was ~1.0 and ~1.2 g/cm^3^, respectively, which is based on the experimentally determined densities for these systems (Hurley and Tzentis, 1963; Vasile and Pascu, 2005).

#### Functionalized SWNTs

**Figure 2:**
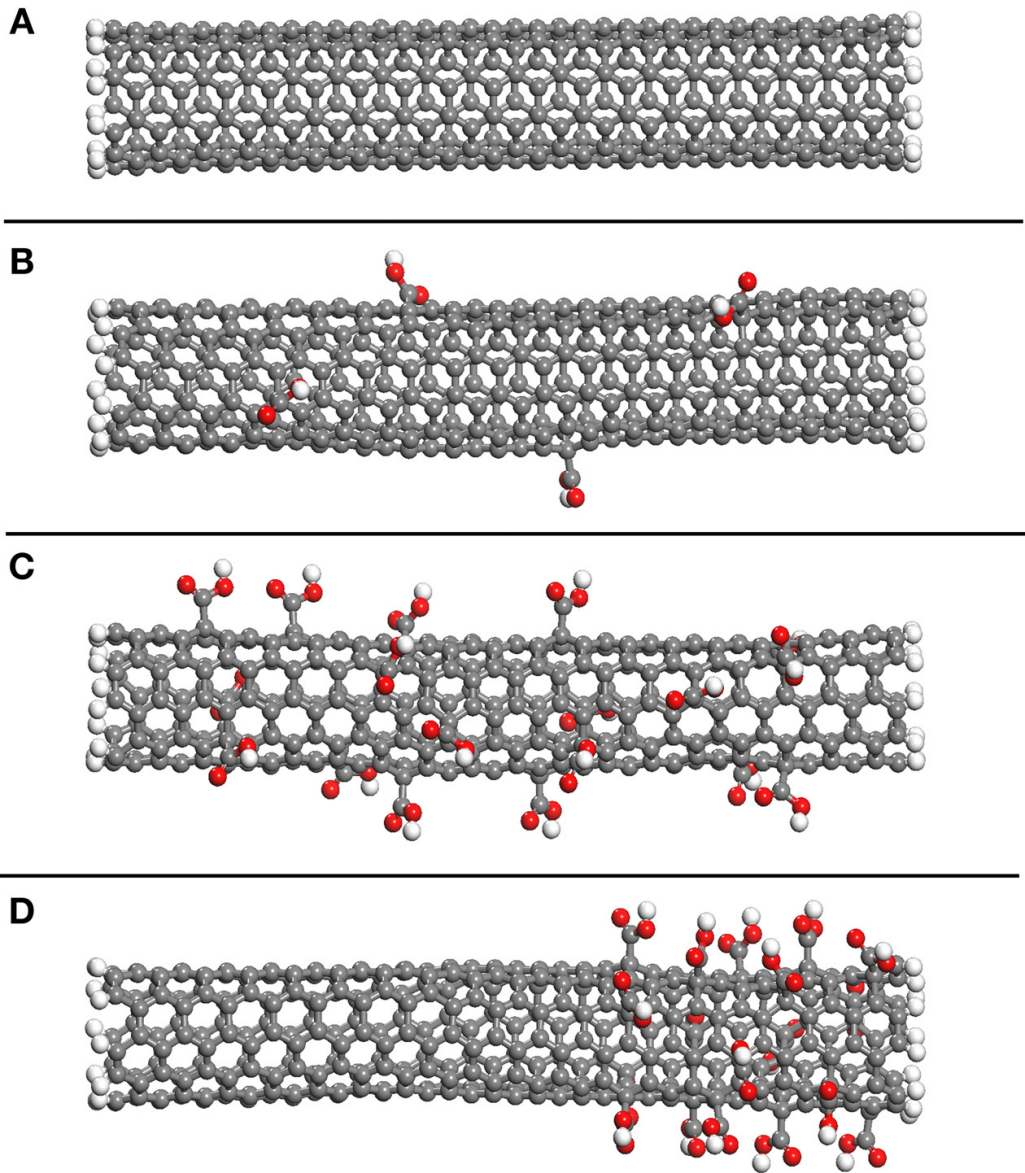
**The non-functionalized SWNT (A), the SWNT with 1% of carbon atoms randomly selected and functionalized with carboxylic acid groups (B), the SWNT with 5% of carbon atoms randomly selected and functionalized with carboxylic acid groups (C) and the SWNT with 5% of carbon atoms near the nanotube end functionalized with carboxylic acid groups (D)**.

Similar periodic cells were constructed using SWNTs functionalized with carboxylic acid groups. The effect of the amount of functionalization was investigated by randomly adding these groups to 1 and 5% of the SWNT carbon atoms, respectively. These values were selected since significant enhancement of the shear stress can be attained for SWNT-polymer systems with 5% functionalization (Zheng et al., 2008). The SWNTs are shown in Figure 2, where Figure 2A shows the non-functionalized SWNT, Figure 2B the SWNT with 1% functionalization and Figure 2C the SWNT with 5% functionalization. Figure 2D shows a SWNT which has 5% functionalization but where all carboxylic acid groups are at one end of the nanotube, and is discussed below. In contrast to the functionalized nanotube in Figure 2D, the carboxylic acid groups in Figures 2B,C are uniformly distributed along the length of the SWNT. As discussed below, this means that the carboxylic acid groups affect the interfacial properties throughout the SWNT pull-out, and not just during a select stage of the pull-out as is the case for the nanotube shown in Figure 2D.

The volume of the SWNT-PE and SWNT-PAN periodic cells were increased to 38.68 Å × 38.68 Å × 71.54 Å and 38.98 Å × 38.98 Å × 71.54 Å, respectively, to prevent interaction between atoms with their periodic images. The number of polymer chains was therefore increased to 101 and 69 chains, respectively, to maintain the same densities as the non-functionalized systems.

The procedure for pulling the SWNT out of the polymer was as follows:

Step 1 (GO): The systems were initially GO in order to decrease the simulation time for equilibration in Step 2. GO was performed until the change in energy between subsequent steps was less than 10^−4^ kcal/mol. Similar to previous studies (Gou et al., 2004, 2005; Zheng et al., 2009; Li et al., 2011; Haghighatpanah and Bolton, 2013), the nanotube was constrained (Jensen, 2007) to allow for the calculations to complete in a tractable time. GO was performed using a combination of the steepest descent (Levitt and Lifson, 1969), conjugate gradient (Fletcher and Reeves, 1964) and Newton (Ermer, 1976) methods.
Step 2 (Equilibration): The structures obtained from Step 1 were used as input for MD simulations in the *NpT* ensemble at 298 K and 1 atm. Equilibration was performed over 100 ps using a 1 fs time step and using the Verlet integration algorithm which has the advantage of being time-reversible (Bolton and Nordholm, 1994). The nanotube structure was constrained during the simulations. The purpose of this step is to generate an initial amorphous polymer structure with low residual stress. The equilibrated structures (seen by constant average total energy and density) had average densities of ~0.9 g/cm^3^ and ~1.1 g/cm^3^ for the SWNT-PE and SWNT-PAN composite structures, respectively, irrespective of the degree of functionalization.

The composite structures were subsequently GO using the same criteria as in Step 1 and as done in previous studies (Gou et al., 2004, 2005; Zheng et al., 2009; Li et al., 2011; Haghighatpanah and Bolton, 2013), and then equilibrated for a further 100 ps in an *NVT* ensemble at 298 K, still maintaining the SWNT constrained. The final equilibration was performed in an *NVT* ensemble at 298 K for 50 ps and 1 fs time step and where the SWNT was no longer constrained. Fluctuations in the temperature and potential energy were ~1% when the SWNT-PE and SWNT-PAN systems reached equilibrium. It may be noted that this equilibration time is sufficiently long since equilibrium—as defined in the previous sentence—was reached after approximately 2 ps.

Step 3 (Pull-out): As in previous investigations (Al-Ostaz et al., 2008; Haghighatpanah and Bolton, 2013), the periodic boundary conditions were removed before the SWNT was extracted from the polymer matrix along the SWNT axial direction. The SWNT was extracted by sequential 4 Å displacements of the SWNT. After each displacement the system was GO to obtain the minimum energy structure. The hydrogen atoms on the SWNT end which was located inside the polymer matrix were fixed to prevent retraction of the SWNT into the matrix during GO. As discussed below, the change in energies of the GO structures during pull-out were used to calculate the interfacial shear stress and interfacial bonding energy. The average results from the three calculations for each system are presented below, and the errors bars are the standard deviations.

### Analysis

For each nanocomposite system, the change in interaction energy at the SWNT—polymer interface was calculated using Equation (1), where *E*_*total*_ is the total potential energy (after GO) of the nanocomposite, *E*_*SWNT*_ is the energy of the nanotube without the polymer and *E*_*polymer*_ is the energy of the polymer without the SWNT.

(1) $$E=E_{total}-\left(E_{SWNT}+E_{polymer}\right)$$

The difference in E between each successive SWNT displacement is the energy increment, Δ*E*. The sum over all Δ*E*, i.e., ∑Δ*E*, is therefore the total energy required to pull the SWNT out of the polymer.

As discussed with reference to **Figure 5** below, and has been observed previously (Li et al., 2011; Haghighatpanah and Bolton, 2013), the average energy increment during Stage II (average Δ*E*_*II*_) is constant and hence independent of the SWNT length. Therefore, the pull-out force can be calculated using Equation (2), where Δ*x* is the displacement increment.

(2) $$F_{pull-out}=\frac{\Delta E_{II}}{\Delta x}$$

The interfacial shear stress, τ, which is the stress when pulling the SWNT through the polymer matrix (Frankland et al., 2002), and the surface energy density, γ, were also calculated to compare with previous results. Two different models have previously been used to calculate τ and γ. Some calculations assume that the interfacial shear stress is uniformly distributed along the length of the embedded CNT (Liao and Li, 2001; Frankland et al., 2002; Ge et al., 2004; Gou et al., 2004; Al-Ostaz et al., 2008; Zheng et al., 2009) (called Model-A below). Other research has reported different locations of maximum τ along the embedded length of the CNT. For example, Natsuki et al. (2007) found that τ is the largest at the preceding end of the CNT. Gao and Li (2005) suggested that the maximum shear stress occurs near both ends of the nanotube, and that the middle of the nanotube is free from shear stress due to symmetry. Li et al. (2011) suggested a similar approach where the τ was distributed solely at each end of the CNT (called Model-B below). This model is used in the present study since it has previously used for similar systems, and the present work analyses whether the two models yield similar trends.

In Model-A, τ is calculated using Equation (3) (Gou et al., 2004) where *r* and *L* are the outer radius and length of the CNT, respectively,

(3) $$\tau_{Model-A}=\frac{E_{pull-out}}{\pi rL^{2}}$$

and where *E*_*pull*−*out*_ = ∑Δ*E*.

In Model-B, τ is calculated using Equation (4) (Li et al., 2011) where *D* is the diameter of the SWNT [6.8Å for the (5,5) SWNT] and *a* is the first and final pull-out stages (8Å, as shown in **Figure 5** below).

(4) $$\tau_{Model-B}=\frac{F_{pull-out}}{2\pi Da}$$

For Model-A, γ is calculated using Equation (5) (Gou et al., 2004) where *E* is the total interaction energy [given by Equation (1)] and *A* is the contact area.

(5) $$\gamma_{Model-A}=\frac{E}{2A}$$

Using Model-B, γ is calculated using Equation (6) (Li et al., 2011).

(6) $$\gamma_{Model-B}=\frac{\Delta E_{II}}{2\pi D\Delta x}$$

## Results and discussion

**Figure 3:**
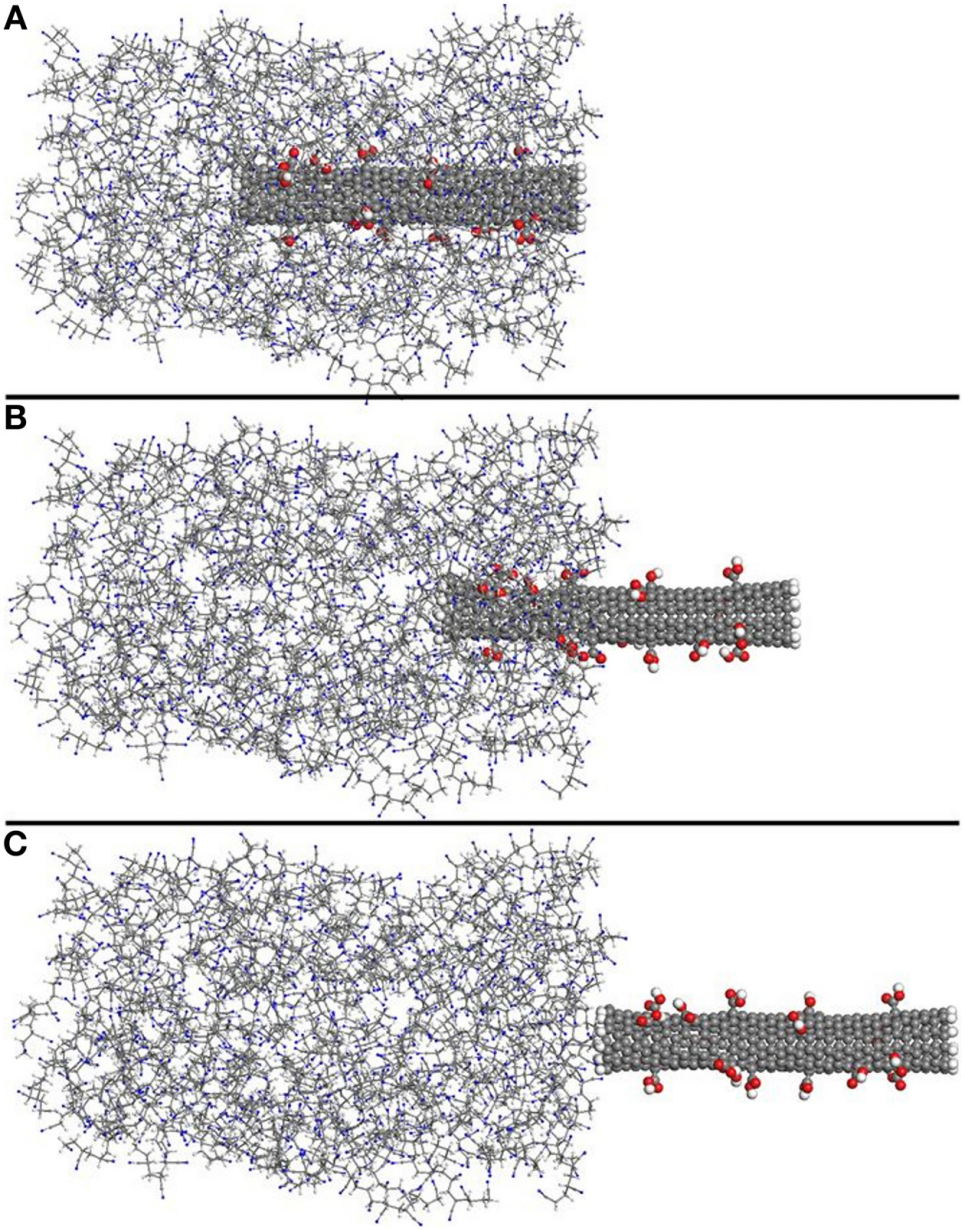
**Typical snapshots during pull-out of the SWNT from the polymer matrix**. The snapshots are for the 5% functionalized SWNT-PAN composite in the initial state **(A)**, at 24 Å displacement of the SWNT **(B)** and at 44 Å displacement of the SWNT where it is completely removed from the polymer matrix **(C)**.

Typical snapshots during pull-out, taken from one of the 5% functionalized SWNT-PAN systems, are shown in Figure 3, where Figure 3A shows the initial state of the nanocomposite at 0 Å displacement of the SWNT, Figure 3B shows the structure at 24 Å displacement and Figure 3C shows the structure at 44 Å displacement of the SWNT (when it is removed from the polymer system).

**Figure 4:**
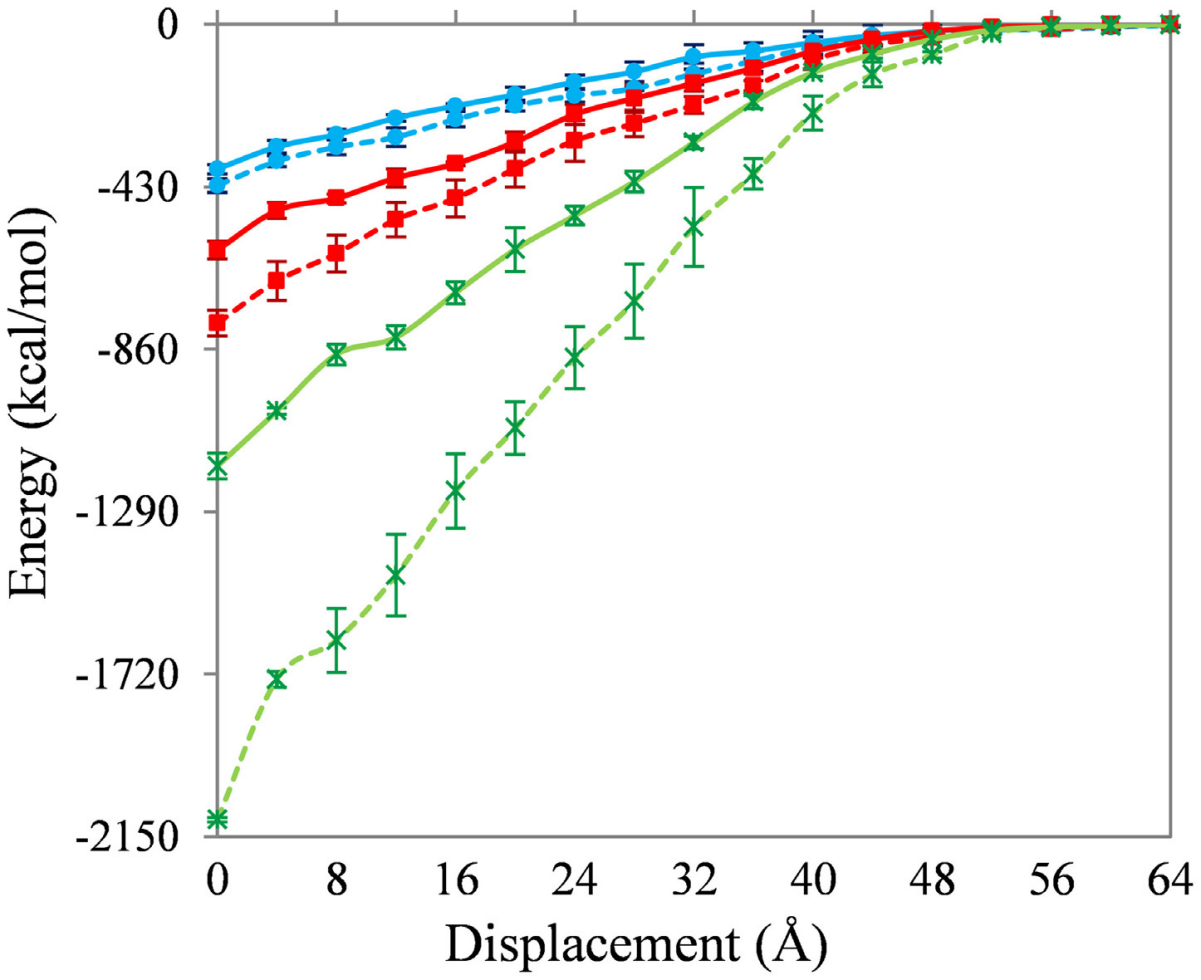
**Change in interaction energy of the SWNT-PE (solid lines) and SWNT-PAN (dashed lines) composites during pull-out**. Results for the non-functionalized, 1% and 5% functionalized SWNT are shown as blue (with filled circles), red (with filled squares), and green (with stars), respectively.

Figure 4 shows the change in interaction energy of the SWNT-PE (solid lines) and SWNT-PAN (dashed lines) composites. The results for the non-functionalized, 1 and 5% functionalized SWNT are shown as blue (with filled circles), red (with filled squares), and green (with stars), respectively. As expected, the interaction energy becomes zero when the nanotubes are completely removed from the polymer matrix.

Three insights are gained from Figure 4. First, the interaction energies between the non-functionalized SWNT and the polymer are similar for the PE and PAN systems. The energy to completely remove the SWNT from the PE matrix (~380 kcal/mol) is only 10% less than to remove the SWNT from the PAN matrix (~420 kcal/mol).

Second, carboxylic acid functionalization of the SWNT leads to a significant increase in the interaction energy, and an increase in functionalization (at least up to 5%) also increases the interaction energy. The energy to completely remove the 1 and 5% functionalized SWNTs from the PE matrix (~600 and ~790 kcal/mol, respectively) is 56 and 205% larger than to remove the non-functionalized SWNT from this matrix. The corresponding values for the PAN matrix (~1170 and ~2100 kcal/mol) are 87 and 397%, respectively. Similar changes in interaction energy during pull-out have been seen previously (Liao and Li, 2001; Niyogi et al., 2002; Gou et al., 2004, 2005; Al-Ostaz et al., 2008; Haghighatpanah and Bolton, 2013), and the energy to completely remove the non-functionalized SWNT from the polymer is also in agreement with previous results (Al-Ostaz et al., 2008; Li et al., 2011; Haghighatpanah and Bolton, 2013) (no results have been reported for the functionalized SWNT-polymer systems studied here). Hence, functionalization with higher coverages of carboxylic acid leads to larger pull-out energies, and the increase with 5% functionalization is approximately four times the increase with 1% functionalization irrespective of the polymer matrix. Similar trends with increasing coverage of functional groups are also found for the other properties discussed below.

Third, functionalizing the SWNT leads to larger changes in the interaction energies for the SWNT-PAN systems than for the SWNT-PE systems. Since the number and location of the carboxylic acid groups is the same in both systems (i.e., the structure of the functionalized SWNTs are the same), this difference is due to the interaction between the carboxylic acid groups and the polymer. This difference is expected since the hydrophilic carboxylic acid groups interact stronger with the polar PAN polymer compared to the PE polymer.

**Figure 5:**
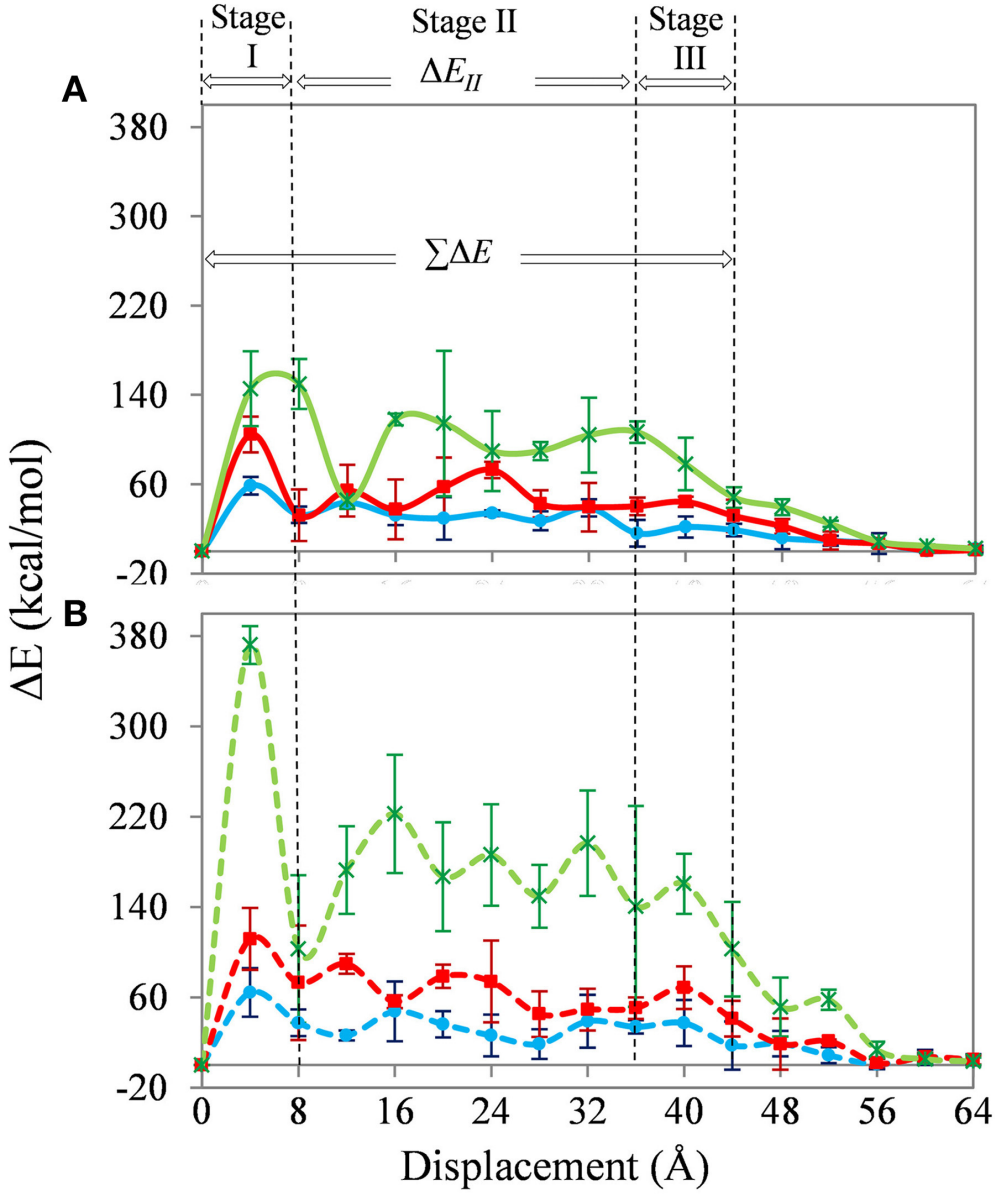
**Energy increments of the SWNT-PE (A) and SWNT-PAN (B) composite systems during SWNT pull-out**. The color code, line types, and symbols are the same as for Figure 4.

The average energy increment, Δ*E*, which is the difference in total energy of the system after each SWNT displacement, is shown in Figure 5. The results for the SWNT-PE and SWNT-PAN systems are shown in Figures 5A,B, respectively. Similarly to previous work (Li et al., 2010, 2011; Haghighatpanah and Bolton, 2013), the changes in Δ*E* are divided into the three stages shown in Figure 5. The largest changes in energy increments for both the SWNT-PE and SWNT-PAN composites are over the first and third stages of the pull-out. In the intermediate stage, Stage II, the increments for SWNT-PE systems fluctuate around a constant value of ~30, ~50, and ~100 kcal/mol when 0, 1, and 5% of SWNT carbon atoms are functionalized, and for the SWNT-PAN systems the corresponding values are ~30, ~70, and ~170 kcal/mol. The fluctuations for the non-functionalized SWNT, which are relatively small, are probably due to the non-uniform coverage of polymer chains along the SWNT walls. As discussed below, the increase in fluctuations for the functionalized SWNTs is due to the interaction between the carboxylic acid groups and the polymer, and depends on the location of the acid groups.

The magnitudes of *F*_*pull*−*out*_ for the SWNT-PE systems are ~80, ~120, and ~260 kcal/(mol nm) when 0, 1, and 5% of the SWNT carbon atoms are functionalized. The corresponding values for the SWNT-PAN systems are ~80, ~160, and ~420 kcal/(mol nm). The results confirm that the force required for pulling the SWNT out of the polymer is higher when the SWNT is functionalized and increases with the amount of functionalization.

The values of ∑Δ*E*, Δ*E*_*II*_, and *F*_*pull*−*out*_ for non-functionalized SWNT-PE systems agree with the values obtained in our previous study (Haghighatpanah and Bolton, 2013), which were ~420 kcal/mol, ~20 kcal/mol, and ~90 kcal/(mol nm), respectively. The values are also in agreement with those obtained by Li et al. (2011), which were ~390 kcal/mol, ~20 kcal/mol and ~90 kcal/(mol nm), respectively. There are no previous results available for the PAN or functionalized SWNT systems that could be used for comparison.

Table 1 shows the values of τ and γ obtained from Model-A and Model-B. The effects of the degree of functionalization and the polymer type are also shown in the table. Three trends can be seen from the table. First, the values of τ and γ obtained from both models show the same trends and are in semi-quantitative agreement. Hence, the choice of model does not affect the conclusions presented here, although it is important to state which model is used when making quantitative comparisons between different calculations.

**Table 1:** **Comparison of τ and γ of SWNT-PE and SWNT-PAN systems with respect to degree of functionalization**.

		**0%**	**1%**	**5%**
**SWNT-PE SYSTEM**				
Model-A	τ (MPa)	150	230	450
	γ (N/m)	0.13	0.19	0.41
Model-B	τ (MPa)	160	240	520
	γ (N/m)	0.12	0.19	0.39
**SWNT-PAN SYSTEM**				
Model-A	τ (MPa)	160	300	820
	γ (N/m)	0.14	0.25	0.67
Model-B	τ (MPa)	170	330	850
	γ (N/m)	0.13	0.26	0.68

Second, the values of τ and γ confirm that the functionalization strengthens the adhesion between the SWNT and polymer matrices, and that a higher load transfer from the polymer to the SWNT can be achieved by carboxylic acid functionalization of the SWNT. Third, the SWNT-PAN system shows higher values of τ and γ, and a larger increase due to the functionalization, compared to the SWNT-PE system, which shows that the effect of the functionalization is larger in the polar matrix.

The value of τ for the non-functionalized SWNT-PE system (from both models) is similar to the value of 141 MPa reported in our previous studies (Haghighatpanah and Bolton, 2013). It differs from the value of 33 MPa reported by Zheng et al. (2009) but is similar to the value of 133 MPa reported by Al-Ostaz et al. (2008) and the value of 142 MPa reported by Li et al. (2011). The value of γ for the non-functionalized SWNT-PE system is similar to the value of 0.14 N/m reported in our previous study (Haghighatpanah and Bolton, 2013), and also agrees with other studies that yielded a range from 0.09 to 0.14 N/m (Lordi and Yao, 2000; Wei, 2006; Li et al., 2011).

The reinforcing effect of the CNTs on the mechanical properties of PE and PAN has also been investigated in several experimental studies (Tang et al., 2003; Weisenberger et al., 2003; Ge et al., 2004; Lozano et al., 2004; Sreekumar et al., 2004; Ye et al., 2004; Chae et al., 2005; Guo et al., 2005; Kanagaraj et al., 2007). However, due to the small scale of CNTs, pullout experiments are hard to perform in practice, and available experimental data are consequently scarce. In addition to the pullout experiments performed by Cooper et al. (2002) and Barber et al. (2003) mentioned above, Ye et al. (2004) observed that SWNTs being pulled out of PAN matrices had no polymer attached to the surfaces of the nanotubes. This indicates a weak interaction between the SWNTs and the PAN matrix, in agreement with the results presented here. MWNTs, however, showed a better interaction with the polymer, and Ye et al. (2004) proposed that this was due to the higher number of surface defects in the MWNTs.

In contrast to the limited number of pullout studies, numerous authors (Tang et al., 2003; Weisenberger et al., 2003; Ge et al., 2004; Lozano et al., 2004; Sreekumar et al., 2004; Ye et al., 2004; Chae et al., 2005; Guo et al., 2005; Kanagaraj et al., 2007) have performed experimental measurements on the effect of CNTs on, for example, the tensile modulus of PE and PAN. All of these studies report improved mechanical properties with the addition of CNTs. For example, Ye et al. (2004) performed tensile tests on PAN-SWNT fibers and the results indicated that the addition of 1% (w/w) SWNTs doubles the tensile modulus.

The reinforcing effect can be further improved by functionalizing the CNTs with COOH groups. Velasco-Santos et al. (2003) measured the tensile modulus of poly (methyl methacrylate) (PMMA) -MWNT composites produced by *in situ* polymerization and where the nanotubes were functionalized with COOH and COO^−^ groups. Scanning electron microscopy (SEM) images revealed an improved interaction between the functionalized nanotubes and the PMMA matrix compared to the unfunctionalized nanotubes. An increase in both maximum stress and strain was achieved with the addition of the functionalized MWNTs compared to the unfunctionalized ones. Kanagaraj et al. (2007) added functionalized nanotubes (e.g., COOH, C = O, and OH groups) to high density PE and obtained a Young's modulus of 1.34 GPa with the addition of 0.44 % (v/v) CNTs (compared to 1.1 GPa for pure HDPE).

The agreement between experimental results and those obtained in the present study, i.e., that increased mechanical properties can be achieved as a result of a stronger polymer-CNT interface, supports the validity of the methods used here. However, it should be noted that there are likely be discrepancies between the quantitative data obtained from experimental and simulation studies, since experimental systems are far more complex (containing impurities, etc.) than the model systems used in simulations. In the present study, the interaction between the CNT and the polymer matrix is due solely to the intermolecular interactions between the defect-free constituents, whereas in a real system CNT defects, covalent linkages between the CNT and the matrix, and alignment of CNT and crystal regions may affect the interfacial shear stress. In this sense, the model presented above yields a valid description of the non-covalent bonding between a SWNT and a PE or PAN matrix. The strength of simulations, compared to many experiments, is that the constituents of the system and their geometries are completely known.

**Figure 6:**
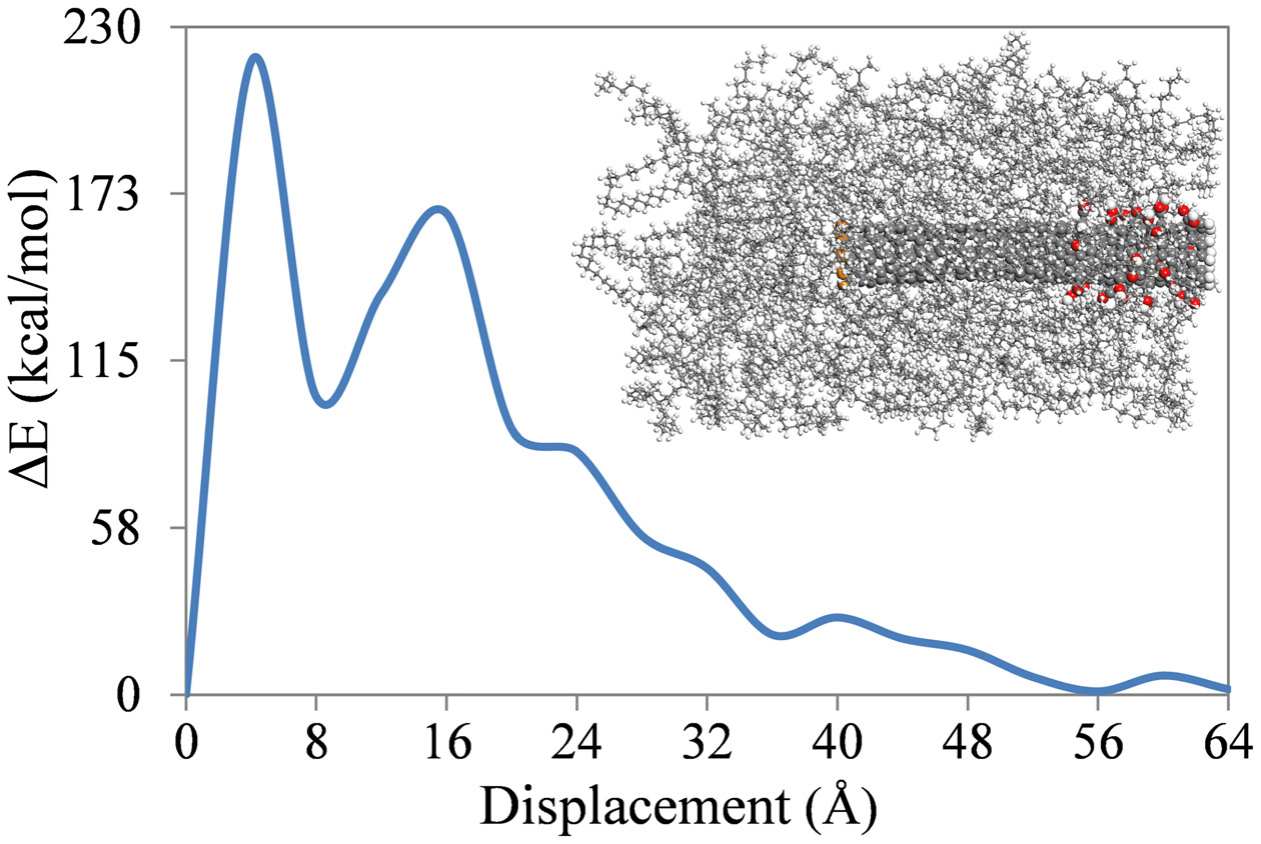
**Energy increments of the SWNT-PE composite during SWNT pull-out where the carboxyl acid groups are located at one end of the SWNT**.

As mentioned with reference to Figure 5, the fluctuations in energy increments in Stage II of pull-out are probably due to the non-uniform distribution of the polymer on the SWNT wall and, in the case of the functionalized SWNTs, are also due to the location of the functional groups on the SWNT. To confirm this, a calculation for the 5% functionalized SWNT in the PE matrix was repeated, but where the functionalization was restricted to one end of the SWNT. This SWNT is shown in Figure 2D and, as shown in the inset to Figure 6, all acid groups are located at the preceding end of the SWNT. As shown in the figure, changes in Δ*E* are significantly different to those shown in Figure 5, where the functional groups were randomly distributed on the SWNT wall. The location of the functional groups at the preceding end of the SWNT results in large energy increments in the initial stage of the pull-out, and then the energy increments decrease to 50 kcal/mol after 28 Å displacement of the SWNT. At this displacement all of the functional groups are out of the polymer matrix, and the energy increments are therefore similar to those for the non-functionalized SWNT (Figure 5). This result emphasizes the importance of the location of the functional groups on the SWNT wall.

It may be noted that the simulations presented here are performed with isotropic, amorphous polymer matrices. Crystalline regions that may form in these composite materials during processing are not considered. If these crystalline regions are far away from the SWNT, then they will not affect the polymer-SWNT load transfer. If the SWNTs act as nucleation sites for the crystalline regions then the load transfer between the crystalline region and the SWNT may differ from the mechanisms studied here (and there may be better transfer is the polymer has adopted a structure based on the SWNT structure). The importance of these crystalline regions on load transfer is left for future studies.

## Conclusion

MM and MD simulations based on the COMPASS force field were used to study the effect of carboxylic acid functionalization on the interfacial bonding characteristics between SWNTs and PE and PAN polymers. This is the first molecular-level computational study of these systems. Functionalization of SWNT carbon atoms, and increasing the extent of functionalization from to 1 and 5%, resulted in larger interaction energies at the interface between the SWNT and polymer matrix. The pull-out simulations showed that, except for the initial and final stages of pull-out, the energy increment fluctuates around a constant average value. The fluctuations depend on the location of the functional groups on the SWNT wall, and increasing the percent of functionalized carbon atoms increases the average energy increment. In addition, the effect of functionalization was larger for the systems containing the polar PAN polymer than for those containing the PE. The larger average energy increments with increased functionalization also results in larger pull-out forces, interfacial shear stresses and surface energy densities. Hence, improved load transfer from PE and PAN polymer matrices to SWNT additives can be obtained by functionalizing the SWNT with carboxylic acid groups, and the improvement is larger for the polar PAN polymer than for PE.

### Conflict of interest statement

The authors declare that the research was conducted in the absence of any commercial or financial relationships that could be construed as a potential conflict of interest.
